# Impact of ivermectin administered for scabies treatment on the prevalence of head lice in Atoifi, Solomon Islands

**DOI:** 10.1371/journal.pntd.0006825

**Published:** 2018-09-25

**Authors:** Suny Coscione, Tommy Esau, Esau Kekeubata, Jason Diau, Rowena Asugeni, David MacLaren, Andrew C. Steer, Christian Kositz, Michael Marks

**Affiliations:** 1 Clinical Research Department, Faculty of Infectious and Tropical Diseases, London School of Hygiene & Tropical Medicine, London, United Kingdom; 2 Atoifi Adventist Hospital, Atoifi, Malaita, Solomon Islands; 3 College of Medicine and Dentistry, James Cook University, Cairns, Queensland, Australia; 4 Centre for International Child Health, University of Melbourne, Department of Paediatrics, Melbourne, Australia; 5 Department of General Medicine, Royal Children’s Hospital, Melbourne, Australia; 6 Hospital for Tropical Diseases, University College London Hospitals NHS Trust, London, United Kingdom; Watford General Hospital, UNITED KINGDOM

## Abstract

**Background:**

Scabies and head lice are ubiquitous ectoparasitic infestations that are common across the Pacific Islands. Ivermectin is an effective treatment for both conditions, although the doses used vary. At a community level, mass drug administration (MDA) with ivermectin is an effective strategy to decrease prevalence of scabies. To what extent MDA with ivermectin will also reduce prevalence of head lice is unknown.

**Methodology:**

Head lice prevalence was assessed before and after MDA with oral ivermectin (at a dose of 200 micrograms per kilogram of body weight) administered on day 1 and day 8. The primary outcome was the change in prevalence of head louse infestation at two weeks compared to baseline. Longer term efficacy was assessed three months after MDA.

**Results:**

118 participants were enrolled. Baseline prevalence of active head louse infestation was 25.4% (95% CI 18.4–34.0). At two-week follow-up, prevalence was 2.5% (95% CI 0.9–7.2), a relative reduction of 89.1% (95% CI 72.7–91.4%, p<0.001). At three-month follow-up, prevalence was 7.5% (95% CI 2.7–12.3), a relative reduction of 70.6% (95% CI 72.7%-91.4%, p <0.001). Head louse infestation was associated with younger age (age ≤10 years: prevalence 46.7%; adjusted odds ratio compared to adults of 7.2, 95%CI 2.0–25.9) and with having at least one other member of the household with active head louse infestation (adjusted odds ratio 4.3, 95%CI 1.7–11.1).

**Conclusions:**

Head louse infestation is common in the Solomon Islands. This proof of principle study shows that oral ivermectin at a dose of 200 micrograms per kilogram can reduce the burden of active head louse infestation, offering an additional collateral benefit of MDA with ivermectin for scabies control.

**Trial registration:**

ClinicalTrials.gov NCT03236168.

## Author summary

Head lice and scabies are both caused by ectoparasites and lead to itchy skin conditions that are associated with secondary bacterial infections and social stigma. Both are common in developing countries. In the Solomon Islands, mass treatment of communities using ivermectin (at dose of 200 micrograms per kilogram) has been shown to be an effective strategy in reducing scabies prevalence. This study shows that the same dose of ivermectin and the same regime of mass drug administration is also effective at reducing the burden of head louse infestation, offering an additional benefit to community wide treatment.

## Introduction

The human head louse, *Pediculus humanus capitis*, is an ectoparasitic insect found on the scalp that is endemic world-wide [1]. Head louse infestation can cause intense scalp itching and scratching can lead to secondary bacterial infections [2]. In low and middle income countries, this parasitic skin disease has received little attention in research [3]. Specifically, in the Pacific region, there is minimal research quantifying its prevalence or potential treatment strategies [4].

Ivermectin is an oral, semi-synthetic derivative of the avermectin family of lactones that selectively binds glutamate-gated chloride channels found in invertebrate muscle and nerve cells thus disrupting neurotransmission in a wide range of human parasites [5]. Oral preparations of ivermectin can be used to treat head lice [2,6] as well as other blood feeding ectoparasitic diseases, including scabies [7,8]. It should be noted that for both, head lice and scabies, oral ivermectin treatment does not have ovicidal action.

A large trial evaluating the use of ivermectin monotherapy, in difficult-to-treat head louse infestation, showed that ivermectin at a dose of 400 micrograms per kilogram (kg) on day 1 and day 8, had superior efficacy compared to 0.5% malathion lotion [5]. Small studies have suggested variable efficacy when a dose of 200 micrograms per kg is used. Single administration resulted in variable reductions in head lice of 45% - 100% at one week follow up but repeat administration on day 8 yielded higher reductions (87.5% - 100%) at two-week follow up [9–13]. These studies have all focused on treatment of individual patients and not on whole communities where head lice are common and where re-infestation from other community members might be anticipated to result in a lower efficacy than that seen in individual treatment trials.

Scabies, another ectoparasitic disease, has already been shown to be extremely common in the Pacific [14–16]. Ivermectin, used as part of mass drug administration (MDA), at a dose of 200 micrograms per kg given at a 7-day interval, has been shown to be an effective strategy to reduce community wide prevalence of scabies in the region [17–20]. These programmes might have ancillary benefits such as treatment of headlice in the communities.

The study we conducted aimed to establish baseline prevalence of head louse infestation in a rural community in the Solomon Islands and to assess whether MDA using ivermectin, at the lower dose of 200 micrograms per kilogram (the same regimen already used for scabies treatment) would be an effective method to lower community prevalence of head lice.

## Methods

### Ethics statement

The study was approved by the London School of Hygiene and Tropical Medicine (LSHTM) ethics committee and the National Health Research and Ethics Committee of the Solomon Islands Ministry of Health and Medical Services. Written consent was obtained from all participants by a staff member fluent in both local languages (Pijin and East Kwaio). Parents or guardians provided written consent for participants aged below 18 years. Children were additionally asked to provide verbal assent for participation. The study was prospectively registered on clinicaltrials.gov (NCT03236168).

### Study Site

The study was conducted in the campus area of Atoifi Adventist Hospital (AAH) in the East Kwaio region of Malaita Island, Solomon Islands. The hospital staff and their families live on campus and create a self-contained community of approximately 200 individuals. The campus has a school for children aged up to 16 years. Students who attend Atoifi School live on campus (staff children) or live in villages that surround the campus. All individuals living on AAH campus and all children and families of children attending Atoifi School were eligible for the study.

### Data collection and ivermectin administration

At baseline participants’ demographic and household data were collected and all individuals underwent a standardised examination of their skin and scalp.

Scalp examination consisted of direct visual inspection of sites of predilection for head lice: the back of the ears, temples and neck. This was followed by parting the hair into 4 sections (first down sagittal plane and then ear to ear). Each section was visually inspected and examined using a metal nit comb with plastic base and 0.1mm teeth size by the brand InfectoPedicul manufactured by InfectoPharm. Eggs and lice were as far as possible not deliberately removed from the hair during the examination to avoid biasing outcome measure assessment by physical delousing. Scalp examinations were carried out by trained nursing staff at Atoifi Hospital led by a physician (SC). Prior to study commencement all staff received a standardised half-day training workshop including practical sessions on clinical examination and use of nit combs. Ten percent of hair examinations were repeated by an independent examiner to ascertain accuracy of diagnosis.

Adult and nymphal stages of head lice were classified as ‘active head louse infestation’. If only head lice eggs were seen, this was recorded as ‘egg infestation’. No attempts were made to differentiate between viable and non-viable eggs.

The diagnosis of scabies was made clinically based on presence of pruritic inflammatory papules or nodules with a typical anatomical distribution using previously validated criteria [21]. The distribution and number of scabies lesions were recorded and used to classify severity of scabies based on classifications used in previous studies in the Pacific region [16,22]. Participants were not re-examined for scabies as part of this study.

Ivermectin was administered as directly observed therapy at a dose of 200 micrograms per kg of body weight. The first administration of ivermectin was at time of baseline examination. A second administration of ivermectin was given 7 days later to all study participants (day 8).

Participants with contraindications to ivermectin treatment (weight below 12.5kg, pregnancy or breastfeeding) were offered malathion 0.5% lotion for head lice and permethrin 5% cream for scabies. Participants diagnosed with any non-scabies skin condition were briefly counselled and, if necessary, advised to attend the medical clinic to obtain treatment as per standard local protocol.

To assess immediate efficacy, a subgroup of participants was re-examined at 48 hours. School children were chosen both for convenience sampling and because they were predicted (from risk factors identified in literature citation) to have highest rates of head louse infestation. The primary outcome of the study was change in prevalence of head lice at 2 weeks compared to baseline. Longer-term efficacy was assessed by examination at 3 months.

### Statistical analysis

For analysis, age was classified into three categories: less than or equal to 10 years, 11–20 years and over or equal to 21 years of age. Area of residence was classified into two categories: household in AAH or household out of AAH campus.

We calculated the prevalence of individuals with adult or nymphal stages of head lice on examination (active head lice) and the prevalence of head lice eggs on examination.

Univariable logistic regression was used to identify risk factors for the presence of head lice at baseline. Variables associated in the univariable regression analysis were included in a multivariable model. We considered age and gender as forced confounders to be included in the multivariable model.

Treatment efficacy was assessed by comparing the proportion of individuals with head lice, using a two-sample test of proportion at baseline compared to 48 hours, two weeks and three months respectively. We calculated the absolute and relative reduction in the prevalence of both active head louse infestation and egg infestation. Data was analysed with STATA software version 14.2 (Stata Corporation, College Station, TX, USA).

## Results

**Fig 1:**
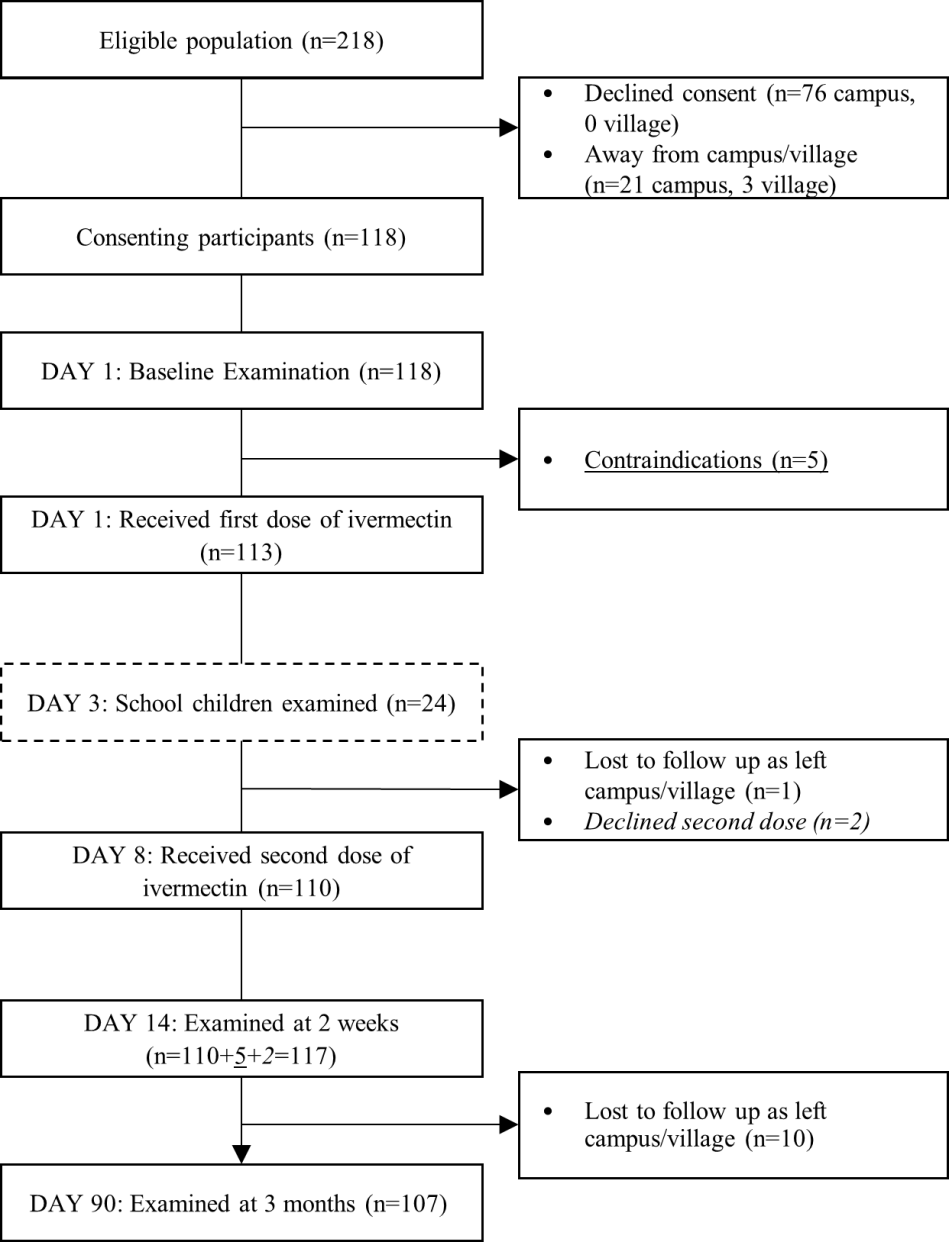
Study enrolment and follow-up flow diagram [modified from CONSORT guidelines [23]].

There were 218 individuals in the study community. Of these, 118 participants consented to take part in the study (Fig 1). Five participants had contraindications to ivermectin (2 women were breast feeding and 3 children weighed less than 12.5kg) and were given malathion lotion and permethrin cream as per protocol.

The median age in the study population was 20 years (interquartile range 10–26). Thirty-seven individuals (31.4% of cohort) lived in one of the three villages closest to AAH campus (Table 1). Households outside of the AAH campus were on average larger and had a larger number of children (3.4) compared to households on the AAH campus (2.4) (p = 0.001).

**Table 1:** Demographic characteristics of study cohort.

	Study cohort (n = 118)
**Gender**	
Male	57 (48.3%)
Female	61 (51.7%)
**Age (years)**	
≤ 10	32 (27.1%)
11–20	31 (26.2%)
≥ 21	55 (46.6%)
**Household location**	
AAH campus	81 (68.6%)
Villages close to AAH	37 (31.4%)

### Baseline prevalence of head lice and scabies

The baseline prevalence of active head louse infestation was 25.4% (95% CI 18.4–34.0). In all cases where active head louse infestation was identified, head lice eggs were also seen. The baseline prevalence of head lice eggs (with or without active head louse infestation) was 42.3% (95% CI 30.8–48.8). Scabies prevalence was 10.2% (95% CI 5.9–16.9) (Table 2). No cases of crusted scabies were identified. Other skin conditions diagnosed included tinea corporis (n = 3) and eczema (n = 4).

**Table 2:** Baseline prevalence of head lice and scabies.

	Active head louse infestation	Head lice eggs	Scabies
Categories	Cases/total in each category	Cases/total in each category	Cases/total in each category
**Total**	30 /118 (25.4%)	50/118 (42.3%)	12/118 (10.2%)
**Age (years)**			
≤10	14/32	21/32	8/32
11–20	12/31	17/31	1/31
≥21	4/55	12/55	3/55
**Gender**			
Male	12/61	17/61	4/61
Female	18/57	33/57	8/57

### Risk factors for head lice

Active head louse infestation was associated with younger age, with increased risk compared to adults for both 0–10 years (AOR 7.2, 95% CI 2.0–25.9, p = 0.003) and 11–20 years (AOR 9.1, 95% CI 2.4–34.2, p = 0.001). Infestation was also associated with the presence of other members of the household with active lice infestation (AOR 3.9, 95% CI 1.1–14.3, p = 0.040). No association was seen with gender or location of the house on or off the AAH campus (Table 3).

**Table 3:** Multivariate analysis of risk factors for head lice.

	Active head louse infestation		Head lice eggs	
	Adjusted Odds ratio (95% CI)	p-value	Adjusted Odds ratio (95% CI)	p-value
**Age (y)**				
≤10	7.2 (2.0–25.9)	0.003	5.9 (2.0–17.1)	0.001
11–20	9.1 (2.4–34.2)	0.001	5.2 (1.8–15.0)	0.002
≥ 21	1.0	-	1.0	-
**Gender**				
Male	1.0	-	1.0	-
Female	1.5 (0.6–4.1)	0.377	3.1 (1.3–7.6)	0.010
**Location of house**				
AAH campus	1.0	-	1.0	-
Villages	1.0 (0.3–3.2)	0.956	1.5 (0.5–4.9)	0.491
**Others infested in household**				
None	1.0		1.0	
At least one person with active head louse infestation	3.9 (1.1–14.3)	0.040	2.2 (0.7–6.7)	0.179

The presence of head lice eggs was associated with age, with highest risk in children aged under or equal to 10 years (AOR 5.9, 95% CI 2.0–17.1). Head lice eggs were also associated with female gender (AOR 3.1, 95% CI 1.3–7.6) (Table 3).

### Change in head lice prevalence at 48 hours, 2 weeks and 3 months

Twenty-four out of 28 school-children (85.7%) were re-examined at 48 hours following ivermectin administration. The prevalence of active infestation decreased from 45.8% at baseline to 0%.

At two weeks after MDA, the prevalence of active infestation had declined significantly (25.6% vs 2.5%, relative reduction (RR) 89.1%, p <0.001) (Table 4).

**Table 4:** Change in prevalence of head louse infestation over 3 months.

Head louse infestation	Baseline prevalence	2-week prevalence	3-month prevalence	Absolute Reduction in Prevalence (baseline to 3 months)	Relative reduction in prevalence (baseline to 3 months)	
	Cases/n (%)	Cases/n (%)	Cases/n (%)	Percentage points (95% CI)	Percentage points (95% CI)	P value
**Active**	30/118 (25.6)	3/117 (2.5)	8/107 (7.5)	18.12 (8.6–27.2)	70.6 (72.7–91.4)	<0.001
**Eggs**	50/118 (42.3)	49/117 (41.8)	21/107 (19.6)	22.7 (11.1–34.4)	53.7 (28.3–70.1)	<0.001

At three months, the prevalence of active head louse infestation remained significantly lower than at baseline (25.6% vs 7.5%, RR 70.6%, p <0.001) (Table 4).

The prevalence of head lice eggs was unchanged at two weeks (42.3% vs 41.8%) but reduced significantly at three months (42.3% vs 19.6%, RR 53.7%, p < 0.001) (Table 4).

## Discussion

This is the first study to demonstrate the efficacy of community-based ivermectin administration for head lice treatment using ivermectin 200 micrograms per kg, administered twice at day 1 and day 8. This regimen significantly reduced prevalence of active head louse infestation at both the two-week and three-month follow-up visit.

At 48 hours, none of the subgroup screened had active head louse infestation. This suggests that the 200 micrograms per kg dose is effective at killing nymphal and adult head lice stages in this population and that a higher dose (400 microgram per kg) is unlikely to offer any additional short-term head lice killing benefits.

At two weeks, the prevalence of active head lice in the whole study sample had decreased significantly (from 25.6% to 2.5%). There was no reduction in head lice eggs at two weeks, however, this was expected given that head lice eggs are cemented, via glue-like glandular discharge, to the hair shaft and remain attached even after hatching. Therefore, head lice eggs will still be found even after the active head louse infestation has been treated.

At three months, the effect of the intervention was sustained. The prevalence of active infestation remained significantly lower than baseline. Furthermore, the reduction in active head louse infestation translated into a reduction in egg prevalence at three months.

Our study also demonstrates that head lice are an extremely common ectoparasitic infection in the Solomon Islands with prevalence comparable or higher than those reported in other lower income settings such as Nepal, Brazil and Egypt [3]. This is a significant burden of disease and further qualitative research is important to elucidate the local attitudes and perception towards head louse infestation.

The findings of this study highlight an opportunity to concomitantly target two common ectoparasitic infections at community level with a single intervention strategy. Showing evidence for additional benefits of MDA may increase engagement of local communities and support from local authorities towards community-wide programs and enables control of head louse infestation to be integrated with growing control programs for neglected tropical diseases such as scabies.

Our study had some limitations. First, MDA coverage was 54% of the eligible population. We did not record the age of non-participants and, as the prevalence of headlice is higher in children, this may have biased our overall prevalence estimate. Despite this our intervention demonstrated efficacy of MDA. Second, prevalence of active head louse infestation did increase slightly at three months compared to two weeks. This may well reflect re-introduction of head lice from untreated members of the community or surrounding villages. Expanding the population that receives treatment may reduce the risk of re-introduction and thus improve long-term reduction in head lice prevalence at community level. Finally, we did not differentiate between viable eggs and non-viable eggs. Being able to reliably distinguish viability of eggs would have yielded a more accurate estimate of active head louse infestation in the community and could be considered in future studies.

Our study demonstrated the effectiveness of a two-stage administration of ivermectin at a dose of 200microgram per kg for the treatment of head lice. A reduction in head lice prevalence is likely to be an ancillary benefit of the scale-up of scabies control programmes in the Pacific region and elsewhere. As with other ectoparasitic infestations, head lice are a common and under-recognised parasitic infestation in the Solomon Islands. Fully measuring the impact of MDA programs can maximise the potential benefits to the community and aid community engagement with the intervention. Further delineating the full range of benefits of ivermectin MDA should be a priority for scabies control programmes.

## Supporting information

S1 Checklist STROBE Checklist. (DOCX)

S1 File Study data. (XLSX)
